# Plant‐made E2 glycoprotein single‐dose vaccine protects pigs against classical swine fever

**DOI:** 10.1111/pbi.12986

**Published:** 2018-08-10

**Authors:** Richard C. Laughlin, Rachel Madera, Yair Peres, Brian R. Berquist, Lihua Wang, Sterling Buist, Yulia Burakova, Sreenath Palle, Chungwon J. Chung, Max V. Rasmussen, Erica Martel, David A. Brake, John G. Neilan, Sara D. Lawhon, L. Garry Adams, Jishu Shi, Sylvain Marcel

**Affiliations:** ^1^ Department of Biological and Health Sciences Texas A&M University Kingsville Kingsville TX USA; ^2^ Department of Anatomy and Physiology Kansas State University Manhattan KS USA; ^3^ iBio CDMO Bryan TX USA; ^4^ U.S. Department of Homeland Security Science and Technology Directorate Plum Island Animal Disease Center Greenport New York USA; ^5^ Oak Ridge Institute for Science and Education Plum Island Animal Disease Center Research Participation Program Oak Ridge TN USA; ^6^ BioQuest Associates LLC Plum Island Animal Disease Center Greenport New York USA; ^7^ Department of Veterinary Pathobiology Texas A&M University College Station TX USA

**Keywords:** veterinary vaccine, classical swine fever, plant‐made pharmaceuticals, DIVA, *Nicotiana benthamiana*, transient expression

## Abstract

Classical Swine Fever Virus (CSFV) causes classical swine fever, a highly contagious hemorrhagic fever affecting both feral and domesticated pigs. Outbreaks of CSF in Europe, Asia, Africa and South America had significant adverse impacts on animal health, food security and the pig industry. The disease is generally contained by prevention of exposure through import restrictions (*e.g*. banning import of live pigs and pork products), localized vaccination programmes and culling of infected or at‐risk animals, often at very high cost. Current CSFV‐modified live virus vaccines are protective, but do not allow differentiation of infected from vaccinated animals (DIVA), a critical aspect of disease surveillance programmes. Alternatively, first‐generation subunit vaccines using the viral protein E2 allow for use of DIVA diagnostic tests, but are slow to induce a protective response, provide limited prevention of vertical transmission and may fail to block viral shedding. CSFV E2 subunit vaccines from a baculovirus/insect cell system have been developed for several vaccination campaigns in Europe and Asia. However, this expression system is considered expensive for a veterinary vaccine and is not ideal for wide‐spread deployment. To address the issues of scalability, cost of production and immunogenicity, we have employed an *Agrobacterium*‐mediated transient expression platform in *Nicotiana benthamiana* and formulated the purified antigen in novel oil‐in‐water emulsion adjuvants. We report the manufacturing of adjuvanted, plant‐made CSFV E2 subunit vaccine. The vaccine provided complete protection in challenged pigs, even after single‐dose vaccination, which was accompanied by strong virus neutralization antibody responses.

## Introduction

Classical Swine Fever (CSF) is a highly contagious hemorrhagic disease affecting pigs. CSF outbreaks have caused substantial economic damages to the swine industry worldwide, including a major outbreak in the Netherlands between 1997 and 1998 that led to the slaughter of over 12 million pigs costing over €2 billion to resolve (Saatkamp *et al*., 2000; Stegeman *et al*., 2000). Classical swine fever virus (CSFV) is an enveloped, single‐stranded (+) RNA virus in the genus *Pestivirus* (Moennig, 2000). The CSFV genome is 12.3 kb and encodes a single polyprotein that is post‐translationally processed into 12 polypeptides by cellular and viral proteases (Lindenbach and Rice, 2007; Rümenapf and Thiel, 2008). These 12 proteins are categorized as structural (C, E^rns^, E1 and E2) or nonstructural (Npro, p7, nonstructural protein (NS) 2, NS3, NS4A, NS4B, NS5A and NS5B). The nonstructural proteins function in viral replication as proteases (Npro, NS2 and NS3), helicase (NS3), or RNA‐dependent RNA polymerase (NS5B) (Lackner *et al*., 2006; Meyers *et al*., 1996).

The virus is highly contagious to both feral and domesticated pigs, and is horizontally transmitted by direct contact of healthy pigs with infected animals (Fritzemeier *et al*., 2000; Laevens *et al*., 1999; Moennig *et al*., 2003). Infection severity can vary widely depending on the virulence of the strain. Clinical signs include: (i) fever, (ii) loss of appetite, (iii) weakness, (iv) conjunctivitis and (v) constipation preceding diarrhoea (World Organization for Animal Health (OIE), 2009), often accompanied by death within 10–20 days of infection (Blome *et al*., 2017; Zaabel *et al*., 2012). As such, CSFV has been designated a Containment Group 4 organism by the OIE and as a United States Department of Agriculture (USDA) Select Agent under the U.S. Federal Select Agent Program (OIE, 2013; Zaabel *et al*., 2012). A neutralizing antibody response against the structural glycoprotein E2 has been detected in pigs that recover from infection and was shown to provide protection against future infection (Hulst *et al*., 1993; König *et al*., 1995; Rümenapf *et al*., 1991). Given the susceptibility of infection across the *Suidae* family, high transmissibility, and lack of palliative care for infected animals, the primary strategies for containing and limiting outbreaks are limiting exposure, culling or vaccination.

Modified live virus (MLV) single‐dose vaccines based on attenuated virus strains are safe and elicit rapid, protective immune responses in naïve pigs, and are commonly used in CSFV endemic areas to protect livestock and contain outbreaks (Blome *et al*., 2006; Van Oirschot, 2003). While providing protection against CSFV infection, animals vaccinated with the most current MLV vaccines are immunologically indistinguishable from wild‐type CSFV‐infected animals. Consequently, pork‐producing countries employing MLV vaccination are negatively impacted by international trade restrictions designed to avoid import of potentially infected pigs into CSFV‐free zones. Thus, a crucial disease control and economic aspect in development of next‐generation CSFV vaccines is to allow for Differentiation of Infected from Vaccinated Animals (DIVA) to support demonstration of freedom from disease.

As an alternative to MLV vaccines, subunit vaccines only require an immunogenic portion of the target virus, permitting vaccine development in lower containment laboratories, and can be designed to develop a DIVA strategy for CSFV control. In addition to DIVA capability, optimally formulated subunit vaccines, can provide additional advantages such as: (i) single‐dose vaccination with long‐lasting protection, (ii) enhanced vaccine stability under field conditions and (iii) low‐cost production. Indeed, CSFV subunit vaccines generated with purified recombinant E2 (expressed in a baculoviral/insect cell system without a transmembrane domain (European Medicine Agency, 2000) are well established and have been licensed for commercial use. However, several product profile limitations have been recognized for the baculovirus‐derived CSFV vaccine including: (i) a slow protective response, (ii) limited protection against vertical transmission and (iii) viral shedding from vaccinates (Blome *et al*., 2017; Zaabel *et al*., 2012). Towards development of an improved CSFV subunit vaccine, Madera *et al*. recently demonstrated protection against CSFV infection in pigs receiving a single dose of a baculoviral/insect cell‐based E2 histidine‐tagged subunit vaccine when formulated in the new KNB adjuvant (Galliher‐Beckley *et al*., 2015; Madera *et al*., 2016). Unfortunately, antigen production using the baculovirus/insect cell expression system remains expensive at manufacturing scale and thus limits wide‐spread deployment of this particular subunit vaccine. With this in mind, we chose to explore an alternative expression platform (*Agrobacterium*‐mediated transient protein production in *Nicotiana benthamiana* plants) that has the potential to significantly reduce the cost to manufacture a novel CSFV E2 subunit vaccine (Nandi *et al*., 2016).

Plants have been used as the production host for recombinant vaccines and other therapeutics for over 25 years (Hiatt *et al*., 1989; Mason *et al*., 1992). Transient protein expression in *N. benthamiana* has become one of the main platforms used to produce plant‐made therapeutics, with manufacturing facilities available to support clinical development and commercial launch (Holtz *et al*., 2015). This platform is well‐adapted to the manufacture of veterinary products because it: (i) is easy to implement, (ii) provides flexible scales of production, (iii) offers rapid development timelines and (iv) has been demonstrated to produce products at lower cost than traditional cell culture‐based expression platforms (Holtz *et al*., 2015; Kolotilin *et al*., 2014; Nandi *et al*., 2016; Shahid and Daniell, 2016). In fact, the first plant‐made veterinary product receiving regulatory approval from the USDA Center for Veterinary Biologics in 2006 was a Newcastle disease virus poultry vaccine (Vermij and Waltz, 2006). CSFV E2 antigen has previously been expressed in and purified from plant cells and was shown to trigger a specific immune response in nontarget animal models (Legocki *et al*., 2005; Marconi *et al*., 2006; Yiu *et al*., 2013) and in pigs (Jung *et al*., 2014). However, no study has been published to date on the protective effect of a plant‐made CSFV E2 antigen against CSFV challenge in pigs. Herein, we describe the design and production of a *N. benthamiana*‐produced recombinant CSFV E2 antigen, and present data assessing immunogenicity, safety and efficacy of the antigen against wild‐type CSFV challenge in pigs.

## Results

### Production of E2 antigens

**Figure 1:**
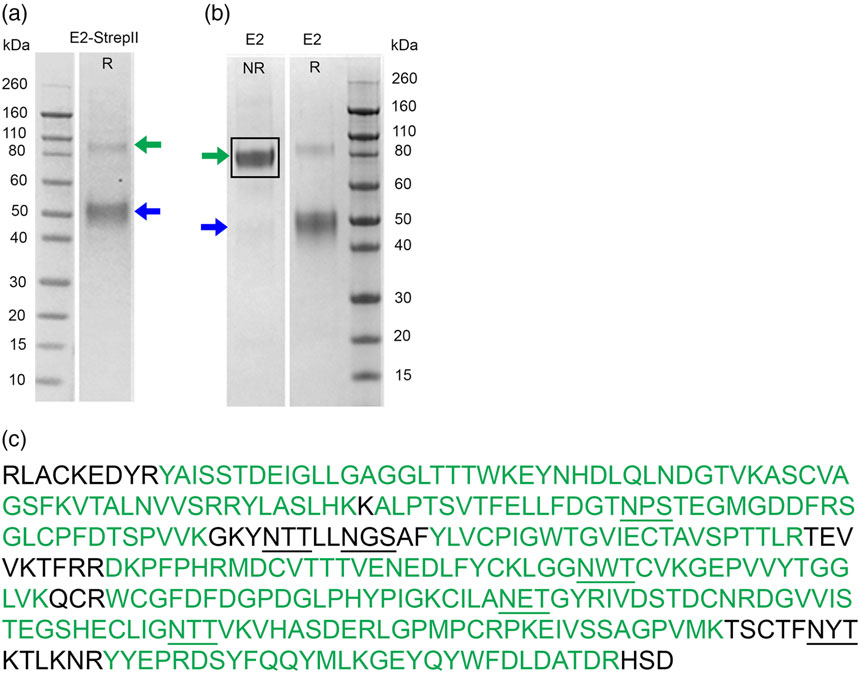
Detection and identity confirmation of untagged E2 soluble protein. (a) SDS‐PAGE of pure E2‐strepII used for the initial immunization study, loaded in reducing condition. (b) SDS‐PAGE of pure untagged E2 protein used for the vaccination/challenge study, loaded in nonreducing (NR) and reducing (R) conditions. All E2 proteins formed a dimer in nonreducing conditions (green arrow) and a monomer in reducing conditions (blue arrow). (c) Results of the LC‐ MS/MS in‐gel tryptic digestion covering 85% of the E2 protein sequence (green sequence). Predicted N‐glycosylation sites are shown in bold and underlined.

Two CSFV E2 antigen vaccine candidates were initially produced: (i) Transmembrane domain‐deleted E2 (GenBank Acc no. ACL98470.1, amino acids 1027‐1063), and (ii) Transmembrane domain‐deleted E2 fused to a StrepII tag (E2‐StrepII). These deletions were made to increase the yield, purity and solubility of the recombinant E2 antigen which are crucial characteristics for easier and economical purification of E2 and vaccine formulation. Also, insect cell‐produced E2 antigens from Porcilis^®^ Pesti (MSD Animal Health, Germany) and Kansas State University (European Medicine Agency, 2000; Madera *et al*., 2016) were also produced as transmembrane domain‐deleted antigens. Both CSFV E2 antigens were expressed in plants by vacuum agroinfiltration and purified by affinity chromatography. The expression of both antigens was estimated to be between 120 and 150 mg/kg of whole plant biomass. The integrity of the antigens was first monitored by SDS‐PAGE (Figure 1). Under reducing conditions, signals at ~50 kDa were detected for untagged E2, consistent with predicted molecular weights of glycosylated monomeric proteins (Figure 1a‐b). Under nonreducing conditions, bands were observed at ~100 kDa for both antigens (untagged E2 and E2‐strepII), consistent with predicted molecular weights of glycosylated dimeric proteins (Figure 1a‐b). Confirmation of E2 dimerization was important as it is a structural attribute of its antigenicity (Thiel *et al*., 1991; Weiland *et al*., 1999). Antigen identity was confirmed by western blot (data not shown), Liquid‐Chromatography Mass Spectrometry (LC‐MS) analysis of intact proteins (data not shown) and tryptic fragmentation of in‐gel isolated protein (Figure 1c).

### Plant‐made E2 induces anti‐E2 IgG responses in pigs

**Figure 2:**
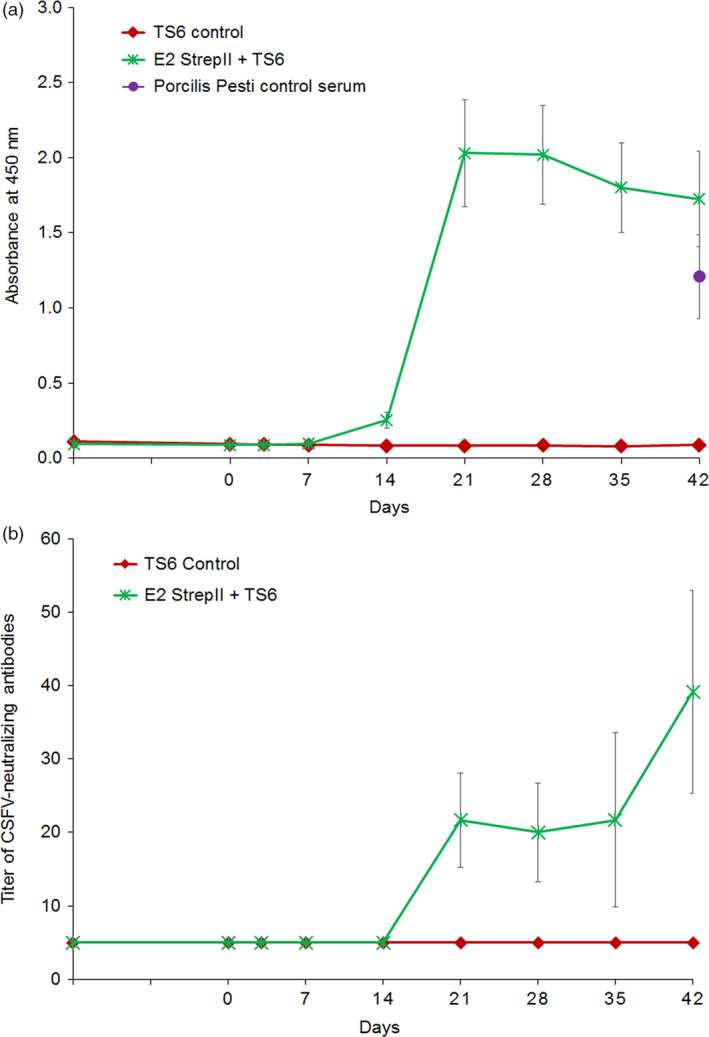
Immunogenicity study of plant‐made CSFV E2 in domestic pigs. Pigs receiving either the E2‐StrepII+TS6 adjuvant, or TS6 adjuvant alone (in PBS) were monitored for E2‐specific immune response by IgG ELISA (a), and CSFV‐neutralizing antibody titres; limit of detection = 5; (b). Error bars: ±SEM.

To determine the safety and immunogenicity of *N. benthamiana*‐produced CSFV E2 administered intramuscularly (IM), naïve pigs were divided into two treatment groups (*n* = 6/group). The first group received 200 μg of E2‐StrepII antigen plus TS6 adjuvant (Boehringer Ingelheim Animal Health, Lyon, France). The second group served as a placebo control (TS6 adjuvant alone). Initial blood samples were taken from each group 14 days prior to vaccination (D‐14) and subsequent blood draws occurred prior to vaccination on the day of vaccination (D0), and after vaccination (D3, D7, D14, D21, D28, D35 and D42). For each group and time‐point, serum generated from whole blood was assayed for E2‐specific IgG by ELISA (Figure 2a, b). Pigs from the first group (E2‐StrepII + TS6) developed anti‐E2 IgG antibodies by 14 days post vaccination (D14) and maintained a high anti‐E2 IgG response throughout D42. This anti‐E2 IgG immune response was comparable to the anti‐E2 IgG response observed in D42 in control serum derived from a pig vaccinated with a commercial baculovirus‐derived recombinant E2 subunit vaccine (Porcilis Pesti^®^, MSD Animal Health). This serum with a characterized anti‐E2‐neutralizing antibody titre (Friedrich Loeffler Institute, Germany) served as a positive control serum in the anti‐E2 IgG ELISA. Serum virus‐neutralizing antibody titres (VNT) measured throughout the study mirrored the anti‐E2 IgG ELISA results demonstrating increasing anti‐CSFV antibody levels over time only in the E2‐strepII‐TS6 group (Figure 2b).

### Plant‐made E2 confers full protection against wild‐type CSFV challenge in pigs

We examined the ability of *N. benthamiana*‐derived CSFV E2 vaccine to confer protection following CSFV exposure. In anticipation of the possible USDA regulatory concerns on use of the genetically fused StrepII tag in the final product, we removed the StrepII tag to produce an untagged, transmembrane deleted, E2 antigen (pE2). We formulated pE2 with one of two adjuvants: (i) TS6 and (ii) KNB (Kansas State University, Manhattan, KS). In addition, efficacy was determined with these pE2 adjuvant formulations and an insect cell‐derived E2 (iE2) KNB‐adjuvanted vaccine, KNB‐E2 (Madera *et al*., 2016). The six treatment groups are summarized in Table 1.

**Table 1:** Treatment groups used for CSFV challenge study

Group	Symbol	Treatment
I	TS6 adjuvant control (Adj/−)	TS6 adjuvant control (Adj), non‐vaccinated, non‐challenged pigs (−)
II	PBS control (−/+)	Challenge control (+), non‐vaccinated challenged pigs (−)
III	TS6‐pE2‐1‐Dose/+	Pigs vaccinated with one dose of TS6 adjuvanted plant‐made E2 vaccine (200 μg antigen) and challenged (+)
IV	TS6‐pE2‐2‐Dose/+	Pigs vaccinated with two doses of TS6 adjuvanted plant‐made E2 vaccine (21 days apart, 200 μg antigen/dose) and challenged (+)
V	KNB‐iE2‐2‐Dose/+	Pigs vaccinated with two doses of KNB‐adjuvanted insect‐derived KNB‐E2 vaccine (21 days apart, 75 μg antigen/dose) and challenged (+)
VI	KNB‐pE2‐2‐Dose/+	Pigs vaccinated with two doses of KNB‐adjuvanted plant‐made E2 antigen formulated with KNB‐E2 emulsion adjuvant (21 days apart, 75 μg antigen/dose) and challenged (+)

**Figure 3:**
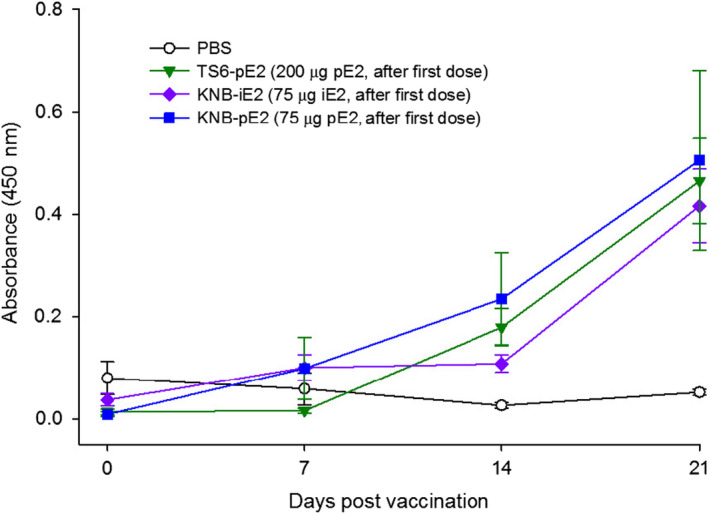
Immune response in pigs after receiving the first dose of vaccination with either the plant‐made E2 (KNB‐pE2 and TS6‐pE2) or insect cell‐made KNB‐E2 (KNB‐iE2) vaccines. Pigs were vaccinated and assessed for levels of E2‐specific IgG prior to CSFV challenge (Day 0 to Day 21). Pig sera were added to the plate at a 1 : 1000 dilution. Error bars: ±SEM.

Pigs from groups III, IV, V and VI had detectable E2‐specific antibodies as early as D14 (post single dose and one week prior to the boost immunization) (Figure 3). Anti‐E2‐specific antibodies generated in pigs immunized with both E2 (groups III, IV and VI) and iE2 (group V) were captured by plant‐made E2 as the coating antigen in the ELISA, confirming the degree of conservation in the immunogenicity profile of both antigens. Interestingly, at D14, anti‐E2 IgG levels from both group IV (TS6‐pE2‐2‐Dose) and group VI (KNB‐pE2‐2‐Dose) were higher than the level from group V (KNB‐iE2‐2‐Dose), though this difference was absent by D21.

**Figure 4:**
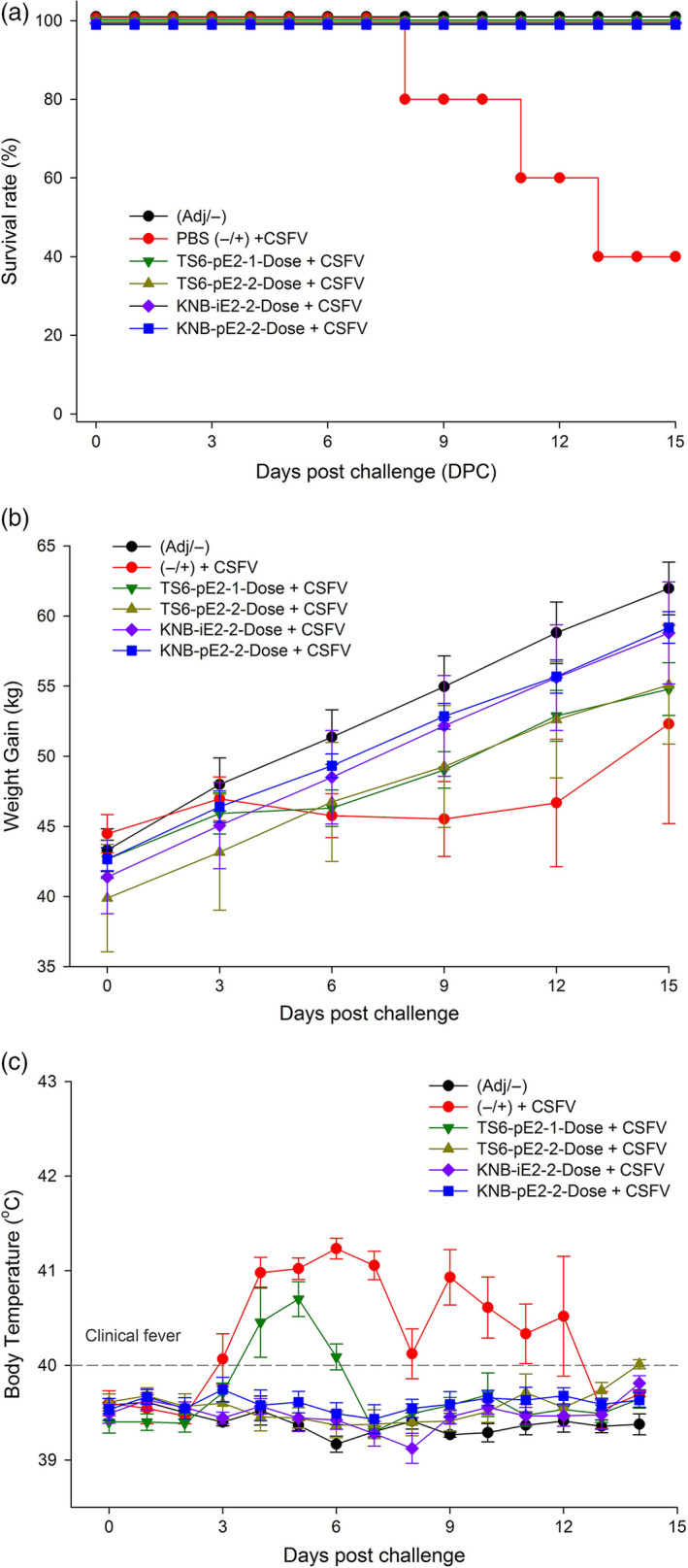
Survival and clinical conditions of vaccinated pigs after challenge. (a) Survival curve of all groups of animals after challenge with wild‐type CSFV, with the death of three pigs from the unvaccinated cohort. (b) Recorded weight over 15 days post challenge. Pigs vaccinated with the TS6‐E2 or the KBN‐E2 formulations showed normal weight gain during the course of the challenge as compared with the unvaccinated control animals. (c) Daily temperatures of vaccinated and naïve pigs after challenge. Error bars: ±SEM.

**Figure 5:**
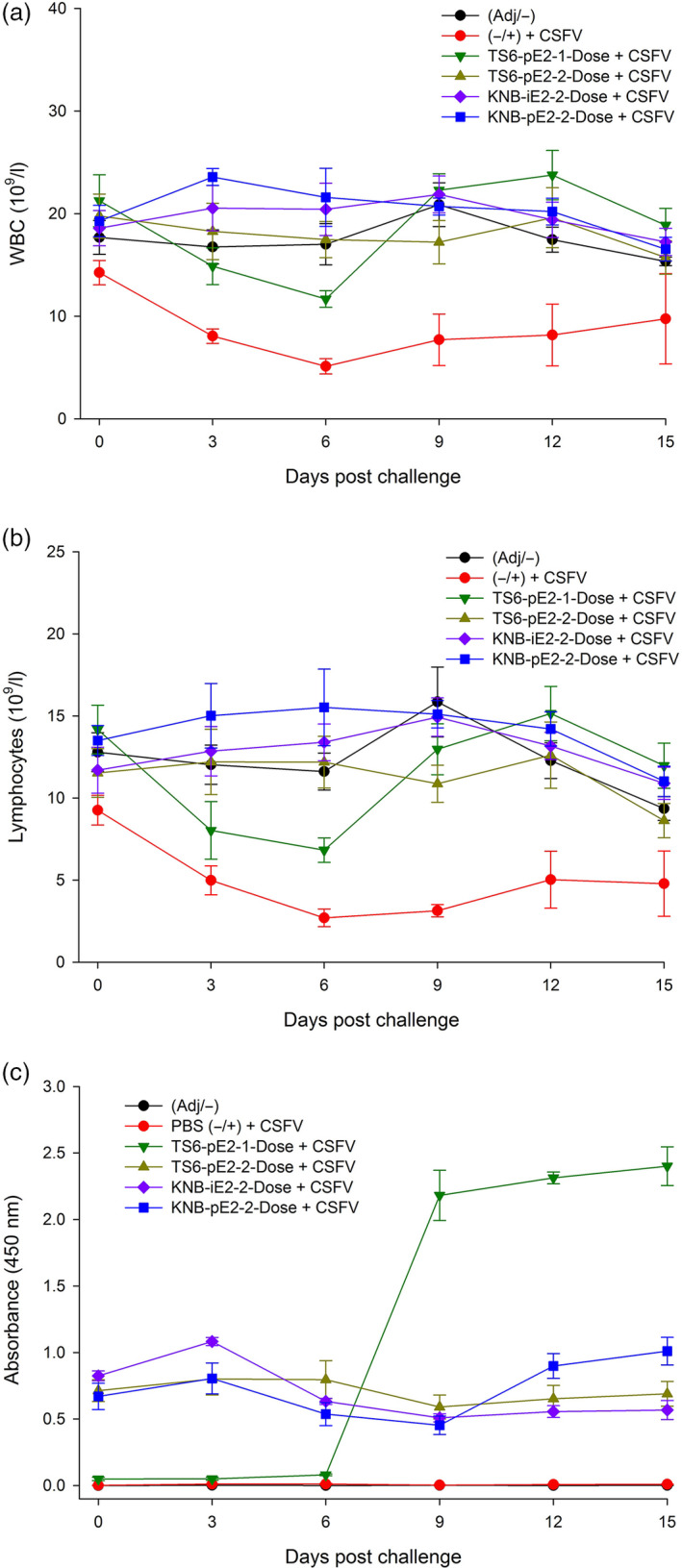
Blood cell count and antibody response in vaccinated animals. (a, b) Pigs vaccinated with E2 subunit vaccine did not develop leucopenia, detected by total white blood cell (WBC) (a) and lymphocyte counts (b). The TS6‐pE2‐1‐Dose vaccinated pigs exhibited transiently lower total WBC and lymphocyte counts and recovered by DPC9. (c) Pigs vaccinated with both plant E2 and insect cell E2 subunit vaccines exhibit high levels of E2‐specific antibody levels. Error bars: ±SEM.

For the CSFV challenge study, treatment groups were transferred to the Kansas State University Biosecurity Research Institute and acclimated for 7 days. On D35 (or Day Post Challenge 0 [DPC0]), each pig was challenged IM with 5 × 10^5^ TCID_50_ (1 mL) CSFV Alfort strain. Body temperature and clinical presentation were monitored up to twice daily for the duration of the study. Three of five pigs from group II (nonvaccinated control) required euthanasia due to the severity of CSF clinical signs at DPC8, 11 and 13 (*e*.*g*. high fever, loss of body weight, diarrhoea, severe leucopenia [±, Figure 4a]). In contrast, all pigs from the vaccinated groups (III, IV, V and VI) survived CSFV challenge (Figure 4a) and continued to gain weight post challenge (Figure 4b). The mean body temperatures of three vaccinated groups (IV, V and VI) remained in normal range (39–40°C) (Figure 4c). However, animals in group III (TS6‐pE2‐1‐Dose) exhibited transiently elevated temperatures (D38‐D40/DPC3‐DPC6), but these resolved by 1 week post challenge and remained in the normal range for the rest of the study. White blood cell (WBC) and lymphocyte counts from vaccinated groups (III, IV, V, VI) remained similar to that of the nonchallenge control group I (Figure 5a‐b). Interestingly, group III (TS6‐pE2‐1‐Dose) showed a brief reduction in cell counts around D38/DPC6, but WBC and lymphocyte levels rebounded to that of groups IV, V and VI prior to D43/DPC9. Taken together, these data provide evidence that both plant‐ and insect cell‐derived E2‐subunit vaccines protected pigs from CSF clinical signs and prevented weight loss, fever and leucopenia following wild‐type CSFV challenge.

### Vaccinated pigs developed anti‐CSFV IgG and neutralizing antibodies before and after CSFV challenge

To assess the antibody response to CSFV challenge, anti‐E2 IgG levels were determined by ELISA. Pigs vaccinated with either plant‐ or insect cell‐derived E2 subunit vaccines (groups III–VI) maintained high levels of E2‐specific antibodies through D50/DPC15 (Figure 5c). Particularly, pigs from group III (TS6‐pE2‐1‐Dose) exhibited a dramatic increase in E2‐specific antibodies between 6 and 9 DPC and maintained high levels through 15 DPC, despite low antibody levels for the first 6 DPC. D41/DPC6 corresponds to the time period that this group of pigs no longer exhibited clinical fever and leucopenia.

Since a crucial factor in preventing CSFV infection/persistence is the production of neutralizing antibodies that block viral infection to target cells, we measured CSFV‐neutralizing antibodies from treated animals in each group (Table 2). All vaccinated groups (III, IV, V and VI) had CSFV‐neutralizing antibodies on the day of challenge (D35) that persisted throughout the postchallenge phase (D50/DPC15). Remarkably, group III (TS6‐pE2‐1‐Dose) displayed the lowest‐neutralizing antibody titres at D35/DPC0, but the highest at D50/DPC15 (DPC15).

**Table 2:** CSFV‐neutralizing antibody titres in pig sera before and after CSFV challenge

Group	Symbol	Pigs	Day 21	DPC0	DPC15
I	TS6 Adjuvant control (Adj/−)	1	(−)	<5	<5
		2	(−)	<5	(−)
		3	(−)	<5	(−)
		4	(−)	<5	(−)
		5	(−)	<5	(−)
II	PBS control (−/+)	1	<5	<5	<5^a^
		2	<5	<5	<5
		3	(−)	<5	<5
		4	(−)	<5	<5^b^
		5	(−)	<5	<5^c^
III	TS6‐pE2‐1‐Dose/+	1	<5	5	>20 480
		2	<5	20	>20 480
		3	20	60	>20 480
		4	15	60	20 480
		5	320	480	20 480
IV	TS6‐pE2‐2‐Dose/+	1	20	1280	10 240
		2	15	7680	5120
		3	(−)	3840	7680
		4	(−)	1920	1920
		5	(−)	3840	3840
V	KNB‐iE2‐2‐Dose/+	1	40	3840	1920
		2	10	3840	2560
		3	80	3840	7680
		4	60	5120	3840
		5	20	3840	2560
VI	KNB‐pE2‐2‐Dose/+	1	60	7680	7680
		2	20	1920	3840
		3	15	2560	5120
		4	7.5	960	10 240
		5	30	1920	7680

Day 21: second dose vaccination day; DPC0 = Day 35; DPC15 = Day 50.

(−): below detection limit; <5: below detection limit (1:5 dilution of serum samples).

a VNT measured at DPC13.

b VNT measured at DPC8.

c VNT measured at DPC11.

Lastly, we sought to assess and quantitate the presence of CSFV in serum and mucosal fluid following challenge. Sera or nasal swabs were used to quantify viral RNA by real‐time, reverse‐transcription PCR. Groups III, IV, V and VI had reduced or undetectable viral loads when compared to Group II (Table 3). In pigs from group III (TS6‐pE2‐1‐Dose) CSFV RNA was detected in the early phase of the challenge (D38/DPC3‐D40/DPC6) before becoming undetectable at D50/DPC15, suggesting early CSFV replication followed by viral clearance by the host immune system. Collectively, these results demonstrated the safety and immunogenicity of a *N. benthamiana*‐derived CSFV E2 subunit vaccine to protect pigs from clinical disease after a virulent CSFV challenge, even after a single dose.

**Table 3:** Detection of CSFV RNA in serum and nasal swab samples from pigs challenged with CSFV

Vaccine groups	Pigs	Ct value – Serum						Ct value – Nasal swab					
		*DPC0*	*DPC3*	*DPC6*	*DPC9*	*DPC12*	*DPC15*	*DPC0*	*DPC3*	*DPC6*	*DPC9*	*DPC12*	*DPC15*
TS6 Adjuvant control (Adj/−)	1	(−)	(−)	(−)	(−)	(−)	(−)	(−)	(−)	(−)	(−)	(−)	(−)
	2	(−)	(−)	(−)	(−)	(−)	(−)	(−)	(−)	(−)	(−)	(−)	(−)
	3	(−)	(−)	(−)	(−)	(−)	(−)	(−)	(−)	(−)	(−)	(−)	(−)
	4	(−)	(−)	(−)	(−)	(−)	(−)	(−)	(−)	(−)	(−)	(−)	(−)
	5	(−)	(−)	(−)	(−)	(−)	(−)	(−)	(−)	(−)	(−)	(−)	(−)
PBS control (−/+)	1	(−)	(−)	(−)	30	24	29	NA	(−)	35	26	25	NA
	2	(−)	(−)	(−)	36	36	(−)	39	(−)	37	35	(−)	(−)
	3	(−)	(−)	39	36	35	35	(−)	39	35	36	38	(−)
	4	(−)	(−)	38	32	NA	NA	NA	38	32	NA	NA	NA
	5	(−)	(−)	36	32	27	NA	NA	36	36	27	NA	NA
TS6‐pE2‐1‐Dose/+	1	(−)	(−)	38	33	36	(−)	(−)	38	38	(−)	38	(−)
	2	(−)	(−)	(−)	37	(−)	(−)	(−)	(−)	38	38	36	(−)
	3	(−)	(−)	(−)	33	(−)	(−)	(−)	(−)	38	(−)	(−)	(−)
	4	(−)	(−)	(−)	33	(−)	(−)	(−)	(−)	35	37	34	(−)
	5	(−)	(−)	(−)	36	(−)	(−)	(−)	(−)	37	37	37	(−)
TS6‐pE2‐2‐Dose/+	1	(−)	(−)	(−)	(−)	(−)	(−)	(−)	(−)	(−)	36	36	(−)
	2	(−)	(−)	(−)	(−)	(−)	(−)	(−)	(−)	(−)	(−)	31	(−)
	3	(−)	(−)	(−)	(−)	(−)	(−)	(−)	(−)	(−)	(−)	36	(−)
	4	(−)	(−)	(−)	(−)	38	(−)	(−)	(−)	(−)	37	34	(−)
	5	(−)	(−)	(−)	(−)	(−)	(−)	(−)	(−)	(−)	(−)	(−)	(−)
KNB‐iE2‐2‐Dose/+	1	(−)	(−)	(−)	(−)	(−)	(−)	(−)	(−)	(−)	38	32	(−)
	2	(−)	(−)	(−)	(−)	(−)	(−)	(−)	(−)	(−)	38	38	(−)
	3	(−)	(−)	(−)	(−)	(−)	(−)	(−)	(−)	(−)	37	32	(−)
	4	(−)	(−)	(−)	(−)	(−)	(−)	(−)	(−)	(−)	36	36	(−)
	5	(−)	(−)	(−)	(−)	(−)	(−)	(−)	(−)	39	38	34	(−)
KNB‐pE2‐2‐Dose/+	1	(−)	(−)	(−)	(−)	(−)	(−)	(−)	(−)	35	36	33	(−)
	2	(−)	(−)	(−)	(−)	(−)	(−)	(−)	(−)	(−)	(−)	37	(−)
	3	(−)	(−)	(−)	(−)	(−)	(−)	(−)	(−)	(−)	(−)	37	(−)
	4	(−)	(−)	(−)	(−)	(−)	(−)	(−)	(−)	(−)	36	37	(−)
	5	(−)	(−)	(−)	(−)	(−)	(−)	(−)	(−)	34	35	33	(−)

(−) below detection limit (Ct value >40); NA, not available because pig was euthanized.

## Discussion

Most MLV CSFV vaccines used in current vaccination programmes provide full protection against clinical disease as early as 5 days post vaccination (Graham *et al*., 2012) but lack DIVA capability. As such, these vaccination programmes have been discontinued in most of Europe (European Commission, 2001) and in other countries declared to be CSFV‐free because of the economic impact related to import/export restrictions on pork products (Marsh *et al*., 2005; Morgan and Prakash, 2006). Because one cannot differentiate infected from vaccinated animals, countries that choose to vaccinate using MLVs have difficulty demonstrating freedom from disease and cannot export pigs or pork products to CSFV‐free countries while the vaccination campaign is ongoing. Therefore, the use of MLV vaccines is often restricted to emergency situations only. In CSFV‐free countries, disease control strategies primarily involve identification of infected animals, slaughter of all pigs within infected areas, movement restrictions and viral surveillance. Outbreaks occasionally re‐emerge in ‘CSFV‐free’ countries, and the expense of control measures deployed by local and national economies becomes extensive when accounting for the costs associated with: (i) depopulation of infected pigs, (ii) preemptive slaughter of currently uninfected pigs in outbreak and neighbouring farms, (iii) movement restrictions, (iv) limited market access, (v) loss of high quality breeding stocks and (vi) ultimately loss of consumer confidence in pigs and pork products from affected areas (Meuwissen *et al*., 1999). It is readily apparent that to achieve CSF disease control and eradication, a vaccine must be DIVA capable. In that regard, CSFV E2 subunit vaccines were developed (Lutticken *et al*., 1998; Moormann *et al*., 2000) and marketed under the brand names Porcilis^®^ Pesti (MSD Animal Health, Germany) and Bayovac CSFV E2^®^ (Bayer, Germany). DIVA diagnostic kits using ELISA for the detection of CSFV E^rns^ antigen were developed and used as companion diagnostic tests alongside the E2‐based subunit vaccine (Meyer *et al*., 2017). Such a diagnostic kit has been successfully evaluated with sera from animals vaccinated with the KNB‐iE2 antigen (Madera *et al*., 2016). Although the original E2 subunit vaccine provided a single‐dose protection, onset of immunity was obtained only after about 10 days (Bouma *et al*., 2000), and two doses were recommended to stimulate longer durations of protection. Nevertheless, development of new subunit vaccines remains a major area of research with the goal of fulfilling the following: early onset of protection with sterile immunity, long‐lasting immunity (the animal life cycle in the pork industry is about 7–8 months), safety without viral shedding among animals and low‐cost production. Importantly, subunit vaccines provide alternatives to protect pig populations against emerging subgenotypes for which current live attenuated CSFV vaccines are not highly protective, such as the emerging subgenotype 2.1d recently reported in India (Gong *et al*., 2016) and south‐east Asia (Luo *et al*., 2017).

In this work, we demonstrated that a *N. benthamiana*‐derived CSFV E2 vaccine protected pigs from a lethal CSFV challenge, even after a single‐dose vaccination as recently demonstrated in pigs vaccinated with insect‐derived E2 formulated with KNB adjuvant (Madera *et al*., 2016, 2018) Robust anti‐E2 IgG and CSFV‐neutralizing antibody responses were generated in vaccinated pigs. Pig groups receiving two doses of E2 vaccine showed no clinical signs, low or no viremia in serum and nasal swabs and continuous weight gain post challenge. Pigs in the single‐dose vaccine dose group exhibited transient fever post challenge, which returned to normal levels 3 days later. This transient fever correlated with transient viremia in blood and nasal swabs. Reduction in fever and viremia in the single‐dose vaccination group (Group III) also correlated with the induction of high anti‐E2 IgG and neutralizing antibodies. In fact, these antibody levels increased dramatically post challenge, beyond levels measured in groups receiving two vaccine doses. One would expect this to occur whenever the challenge virus replicates and induces a fever response. Future work will closely examine the early onset of protection and viral shedding, typical criteria for evaluating complete protection in vaccinated pigs. Evaluation of those two aspects will be particularly important in an emergency situation where viral replication needs to be fully controlled and eliminated quickly.

Full protection was also achieved with commercial E2 subunit vaccines at 14 days post vaccination, but offered only partial protection before 10 days post vaccination (Bouma *et al*., 2000; Uttenthal *et al*., 2001). However, these vaccines were offered as 32 μg of antigen per dose at >$2.50/dose (personal communication). Although it is possible that a higher dose could provide better protection, the cost per dose would likely become prohibitive for the end‐user. In contrast, the *N. benthamiana* plant expression system offers extra flexibility and represents a more cost‐effective alternative which can provide additional antigen per dose for a fraction of the cost of bioreactor‐based manufacturing methods (Nandi *et al*., 2016). Indeed, the reduced cost of goods manufactured using the *N. benthamiana* transient platform is primarily attributed to a simple and low‐maintenance upstream process, easy scale‐up of production and faster development time due to the transient nature of the expression system (Holtz *et al*., 2015; Nandi *et al*., 2016). Thus, it is anticipated that the *N. benthamiana* plant expression system will provide single‐dose DIVA‐capable CSFV subunit vaccines with a lower manufacturing cost of goods. Alternatively, and to reduce manufacturing costs further, an edible vaccine formulation using non‐ or semi‐processed plant tissue expressing the E2 antigen may be tested (Merlin *et al*., 2017; ProVacs‐Production of Vaccines from Applied Crop Sciences, 2005). In fact, oral bait formulations of E2 antigen have been tested in Europe with some success in controlling CSF in the wild boar population (Rossi *et al*., 2015) and can be revisited with an improved process and formulation. Additional process evaluation and cost analysis will be needed to define the potential cost savings of using the plant expression platform for new CSFV vaccine candidates. In conclusion, this study demonstrated the safety, immunogenicity, efficacy and potentially low‐cost scalability of a single‐dose plant‐based CSFV E2 vaccine.

## Experimental procedures

### Cloning and expression of recombinant E2 antigen

The genetic sequence of the CSFV E2 protein C‐strain‐ZJ (GenBank Acc # ACL98470.1, amino acids 689‐1026 with E782G substitution) fused to the signal peptide of the barley α‐amylase (GenBank Acc # CAX51374.1) was codon optimized for expression in *N. benthamiana* plants (ATUM, Newark, CA). A second gene (E2‐StrepII) was prepared with the fusion of the StrepII tag sequence WSHPQFEK on the C‐terminus of the E2 sequence. All genes were inserted into a Tobacco Mosaic Virus‐based proprietary expression vector (iBio Inc., New York, NY) and mobilized in *E. coli* 10G ELITE electrocompetent cells (Lucigen, Middleton, WI) by electroporation for vector amplification. After sequencing of the insert, expression vectors were mobilized into *Agrobacterium tumefaciens* strain GV3101 together with the pSOUP helper vector (Hellens *et al*., 2000) and cultured at 28 °C, 225 rpm in Luria‐Bertani media supplemented with 50 mg/L kanamycin, 25 mg/L gentamycin and 25 mg/L rifampicin. Transient expression of recombinant E2 proteins was performed by vacuum infiltration as described previously (Holtz *et al*., 2015). Briefly, *N. benthamiana* seeds were germinated on rockwool Kiem^®^ plugs (Grodan, Roermond, The Netherlands) placed in Sure To Grow hydroponic media (Sure To Grow, Beachwood, OH) at ~26°C under a 16/8 h LED light/dark photoperiod. Five‐week‐old *N. benthamiana* plants were vacuum infiltrated for 3 min at 23 in. (584 mm) Hg in 5 mm MES buffer, pH 5.5 containing overnight cultures of Agrobacteria diluted to a final OD_600 nm_ of 0.05. Infiltrated plants were placed in a growth chamber under constant light at approximately 24 °C.

### Purification of recombinant E2

The plant‐derived E2 antigen was produced in *N. benthamiana* plants. After 7–8 days post infiltration (DPI), plants were harvested and homogenized in two volumes (w:v) of cold buffer (50 mm sodium phosphate, 150 mm sodium chloride, 70 mm ascorbic acid, 5 mm EDTA, pH 8.0) and centrifuged at 14 000 ***g*** for 15 min at 12 °C. Major host cell proteins were precipitated by adjusting the pH of the extract to 4.8 with 2 m acetic acid. After a 2‐min incubation, the pH of the extract was adjusted to pH 7–8. The extract was centrifuged again at 14 000 ***g*** for 15 min at 12 °C and clarified through a 0.2 μm Sartopure PP3, size 4 filter (Sartorius, Bohemia, NY). For E2‐StrepII antigen purification, extracts were loaded on 5 mL StrepTrap HP column (GE HealthCare Life Sciences, Piscataway, NJ) following the manufacturer's instructions. For untagged E2 antigen purification, clarified extract was loaded on a HiTrap NHS‐Activated HP Sepharose column (GE HealthCare Life Sciences, Piscataway, NJ) coupled with the anti‐E2 antibody WH303 (APHA, Addlestone, UK). Plant‐made E2 was eluted from the immunoaffinity column with 5 CV of 100 mm citric acid. Elution fractions were then dialysed against 1X Phosphate‐buffered saline (PBS), pH 7.4 overnight at 4 °C. The insect cell‐derived E2 was produced and purified as described previously (Madera *et al*., 2016).

### SDS‐PAGE and western blotting

Samples collected during processing and pure E2 proteins were analysed by SDS‐PAGE on 4%–10% Bis‐Tris gradient NuPAGE^®^ gel (Life Technologies, Carlsbad, CA). Samples were reduced with 2.5% β‐mercaptoethanol. Proteins were either stained with SimplyBlue SafeStain (Life Technologies, Carlsbad, CA) or transferred to a nitrocellulose membrane for western blotting. Western blot membranes were first blocked 1 h with 3% bovine serum albumin (BSA) in Tris‐buffered Saline (TBS) supplemented with 0.5% Tween‐20 (TBST). The membrane was then probed for 1 h with WH303 anti‐E2 antibody (APHA Scientific, Addlestone, UK) used at 1 : 10 000 dilution in TBST, 1% BSA. After three successive washes with TBST, the membrane was probed for 1 h with alkaline phosphatase‐labelled goat anti‐mouse antibody (Sigma‐Aldrich, Saint Louis, MO) used at 1 : 5000 in TBST. Membranes were developed with NBT/BCIP substrate (Sigma‐Aldrich, Saint Louis, MO). Stained gels and western blot membranes were imaged using a Bio‐Rad Gel Doc™ XR+ imager (Bio‐Rad, Hercules, CA).

### Ethics statement

All animal experiments were conducted following research protocols approved by the Institutional Animal Care and Use Committees (IACUC) of Texas A&M University and Kansas State University.

### Pig immunogenicity study (College Station, TX)

The animal immunization study was performed at the Texas A&M Veterinary Medical Park under the approval of Texas A&M University IACUC protocol 2015‐0271. Twelve (12) Yorkshire‐Hampshire cross‐bred 4‐week‐old pigs weighing 18 kg each were purchased from an approved commercial vendor and were housed under standard conditions. Pigs were porcine circovirus 2 (PCV‐2) and PRRSV free or vaccinated (Fostera^®^ PRRS, Zoetis, Kalamazoo, MI) 2 weeks prior to shipment. Pigs were also vaccinated with Swine influenza virus and *Mycoplasma hyopneumoniae* (FluSure XP^®^/RespiSure^®^, Zoetis, Kalamazoo, MI), *Bordetella bronchiseptica*,* Actinobacillus pleuropneumoniae*,* Haemophilus parasuis*,* Erysipelothrix rhusiopathiae*,* Streptococcus suis*,* Pasteurella multocida* (Parapleuro Shield P+BE, Elanco, Larchwood, IA) and Leptospirosis (Lepto Shield 5, Elanco, Larchwood, IA) 2 weeks prior to shipment. Pigs were acclimated for 2 weeks prior to beginning of the vaccine study. Pigs were randomly grouped and inoculated intramuscularly (IM) with either 200 μg of plant‐made CSFV E2 antigen adjuvanted with TS6 (Boehringer Ingelheim Animal Health, Lyon, France), or TS6 adjuvant alone (mixed with PBS). An IM booster dose was administrated 28 days after the first injection. Serum was collected on DAY (‐)14, 0, 7, 14, 21 28, 35 and 42 days to determine the level of antibody response to the treatment. Safety concerns were assessed by daily monitoring of pigs during the first 7 days after immunization by a veterinarian. Injection site lesions, animal behaviour and weight were also recorded.

### Quantification of anti‐E2 antibodies in pigs from immunization study

Anti‐E2 antibodies (IgG) were measured in E2‐vaccinated pig sera by ELISA. Briefly, 40 ng/mL of purified plant‐made untagged E2 prepared in PBS was coated overnight at 4 °C in 96‐well flat‐bottomed microtiter NUNC Maxisorp plates (ThermoFisher, Waltham, MA). The untagged E2 protein was used in the ELISA set up to avoid the detection of antibodies raised against the StrepII tag during the pig immunization. Plates were washed four times with PBS + 0.5% Tween‐20 (PBST) and wells were blocked with 5% nonfat dried milk in PBST for 1.5 h at 37 °C. Plates were washed four times with PBST. Then, diluted sera (samples run in duplicate) were added and plates were incubated for 1 h at 37°C. Plates were washed four times with PBST before horseradish peroxidase (HRP)‐conjugated goat anti‐porcine IgG (Bethyl, Montgomery, TX) was applied. Plates were developed with 3,3,5,5 tetramethylbenzidine (TMB) substrate (Bethyl, Montgomery, TX), and the reactions were stopped with 1 N hydrochloric acid. Relative antibody concentrations were calculated from optical spectrophotometer readings at 450 nm using a Biotek Synergy H1 hybrid microplate reader and analysed with Gen5 Data Analysis Software (Biotek, Winooski, VT). A positive control, serum collected at day 42 from a pig vaccinated with MSD Porcilis Pesti^®^ subunit vaccine, was kindly provided by Dr. Sandra Blome, Friedrich Loeffler Institute, Greifswald, Germany and served as a reference in each ELISA plate.

### Serum anti‐CSFV neutralization antibody assay (immunization study)

The virus neutralization test (VNT) to determine neutralizing antibody titre in serum samples was carried out as previously described (Chen *et al*., 2010). Briefly, 50 μL of 100 TCID_50_ CSFV Brescia strain were mixed with an equal volume of twofold serially diluted pig sera in a 96‐well plate and incubated at 37 °C for 1 h before 100 μL of DMEM containing 1 × 10^5^/mL SK6 cell, 10% FBS and 1X antibiotic‐antimycotic was added to all wells in 96‐well plates. Serum samples were diluted in series starting from 1 : 5 to 1 : 10 240. At 96 h post inoculation, cells were fixed with 100 μL/well of chilled 50 : 50 acetone/methanol. Immunocytochemical staining of CSFV in cells with commercial anti‐E2 antibody (WH303 clone, APHA, Addlestone, UK) and VECTASTAIN Elite ABC HRP Kit (Vector lab, Burlingame, CA; PK‐6102) were done per manufacturer's instructions. Neutralizing antibody titres in serum samples were expressed as the reciprocal of the highest dilution that caused 50% neutralization. Due to unavailability of C‐strain‐ZJ CSFV used for recombinant vaccine design, the CSFV Brescia strain, with amino acid sequence identity and homology (91.4% and 94.9%), was used in this VNT. Therefore, VNT titres in this assay may be a more conservative estimate than analysis using the autologous strain.

### Challenge study (Manhattan, KS)

All animal studies were performed under the Kansas State University (KSU) IACUC approval (IACUC# 3845). Conventional Large White‐Duroc cross‐bred weaned specific pathogen‐free 3‐week‐old female piglets were obtained from a commercial source. The pigs were kept in a biosafety level II (BSL2) laboratory at the Kansas State University Large Animal Research Center (LARC) prior to CSFV challenge. For the CSFV challenge phase, pigs were transferred to the biosafety level III Agriculture laboratory at the Biosecurity Research Institute (BRI, Kansas State University). Naive pigs were randomly assigned into six groups of five pigs each (Table 1). The plant‐made E2 protein was obtained from iBio CDMO and the transmembrane domain‐deleted insect cell‐derived E2 was expressed and purified in KSU laboratory as described previously (Madera *et al*., 2016). The different CSFV E2 subunit vaccine formulations were prepared by mixing the purified antigen with the adjuvant. Antigen formulations and controls were administered IM. Two groups of pigs received either one or two doses of plant‐made E2 antigen (pE2) formulated with the TS6 oil‐in‐water emulsion (200 μg of antigen per dose in 1 mL). Group III (TS6‐pE2‐1‐Dose) received a single dose of pE2 plus TS6 adjuvant on D0, and Group IV (TS6‐pE2‐2‐Dose) received a priming dose of 200 μg pE2 plus TS6 adjuvant on D0, a booster dose on D21. Group V (KNB‐iE2‐2‐Dose) received a priming dose of 75 μg of insect cell‐derived E2 antigen (iE2) in the KNB oil‐in‐water emulsion (75 μg of antigen per dose in 2 mL) on D0, and a booster dose on D21. Group VI (KNB‐pE2‐2‐Dose) received two doses of 75 μg of pE2 plus KNB adjuvant, one on D0 and one on D21. Group I (Adj/−), the placebo control, received 2 mL of TS6 adjuvant mixed in PBS (Phosphate‐Buffered Saline) on D0, and were the only group of pigs not challenged with CSFV. Group II (−/+) served as a nonvaccinated control, received 2 mL of PBS alone on D0. On Day 35, pigs in Groups II–VI were challenged with 5 × 10^5^ TCID_50_ CSFV isolate Alfort (1 mL IM). Pigs clinical signs, weight and rectal temperatures were monitored every day. Sera were sampled on Day 0 (day of first vaccination) and Day 21 (day of second vaccination or 21 DPV). Whole blood, serum and nasal swabs were sampled on Day 35 (day of CSFV challenge or 35DPV/DPC0) and every 3 days after that. Total white blood cell (WBC) and leucocyte differentiation counts were carried out as described before (Madera *et al*., 2018).

### Quantification of anti‐E2 antibodies in challenged pigs (challenge study)

Anti‐E2 antibodies (IgG) were determined in pig sera by ELISA. 96‐well flat‐bottomed microtiter plates (Corning, Corning, NY) were coated with 62.5 ng/mL of purified plant‐made untagged E2. The untagged E2 protein was used in the ELISA set up to avoid the detection of antibodies raised against the StrepII tag during the pig immunization. Diluted sera (samples run in duplicate) were added and plates were incubated for 1 h at 21 °C. Goat anti‐porcine IgG labelled with horseradish peroxidase was used as secondary antibody (Southern Biotech, Birmingham, AL). The ELISA plates were developed with 3,3,5,5 tetramethylbenzidine (TMB) substrate (Life Technologies, Carlsbad, CA). The reactions were stopped with 2 N sulphuric acid before plates were read at 450 nm using a SpectraMAX microplate reader. Relative antibody concentrations were then calculated using the Softmax^®^ Pro 6.4 Software (Molecular Devices, Sunnyvale, CA).

### Virus quantification in sera and nasal swabs by RNA isolation and real‐time RT‐PCR (challenge study)

Viral RNA was isolated from sera and nasal swabs using the IBI Viral Nucleic Acid Extraction Kit II (IBI Scientific, Peosta, IA) following the manufacturer's instructions. Real‐time RT‐PCR was performed using CSFV‐specific primers as previously described (Hoffmann *et al*., 2005). For quantification, a standard curve was prepared using serial dilutions (10^7^–10^2^) of a passage 4 CSFV stock (10^7^ TCID_50_) before viral RNA isolation. Viremia was calculated using StepOne™ Software v2.3 (Applied Biosystems, Foster City, CA).

### Serum anti‐CSFV neutralization assay (challenge study)

A neutralization assay using indirect fluorescent antibody assay (IFA) as the readout method was performed to calculate the anti‐CSFV‐neutralizing titres in CSFV‐challenged pig sera as described previously (Madera *et al*., 2018). Neutralizing antibody titres in serum samples were expressed as the reciprocal of the highest dilution that cause 50% neutralization.

## Conflict of Interest

Y. Peres, B. R. Berquist, S. Palle and S. Marcel are employed by iBio CDMO. The other authors declare no conflict of interest.
